# Effect of Low-Level Laser Therapy on Knee Range of Motion and Functional Abilities After Total Knee Arthroplasty

**DOI:** 10.7759/cureus.50893

**Published:** 2023-12-21

**Authors:** Bandar A Alghamdi, Rania N Karkousha, Adham A Elgeidi, Fatma S Amin, Ahmed M Tolba

**Affiliations:** 1 Orthopedic, Department of Surgery, College of Medicine, Umm Al-Qura University, Al-Qunfudhah, SAU; 2 Physical Therapy, Basic Science Department, Faculty of Physical Therapy, Cairo University, Cairo, EGY; 3 Orthopedic and Traumatology, Orthopedic Department, Faculty of Medicine, Mansoura University, Mansoura, EGY; 4 Physical Therapy, Basic Science Department, Faculty of Physical Therapy, Delta University for Science and Technology, Gamsa, EGY

**Keywords:** total knee replacement, osteoarthritis, range of motion, rehabilitation, low level laser, functional abilities

## Abstract

Objective: The aim of this study was to determine the effectiveness of combined low-level laser therapy (LLL) and rehabilitation in patients following recent total knee replacement (TKR).

Methods: A double-blind randomized controlled study was conducted at the Orthopedic Department of Mansoura University Hospital. Forty-four patients were chosen from a total of 58 patients who met the inclusion criteria and were assigned randomly into control and experimental groups of equal size. Ultimately, 40 patients completed the study (20 from each group). Both groups participated in an intensive functional rehabilitation program, and the experimental group also received LLL therapy around the knee at the incisional line, the medial and lateral intra-articular space, above and below the patella, and at the popliteal fossa at low fluence (6 J/cm^2^, 650 nm continuous wave) and 60 s per point with a total dose of 48 J per session over 12 treatment sessions for six weeks. Knee range of motion (ROM) was measured with a digital goniometer, and functional abilities were assessed with the Arabic version of the Western Ontario and McMaster Universities Osteoarthritis (WOMAC) index.

Results: There were significant differences in all variables pre- and post-treatment within each group. Before treatment, there was no significant difference in any of the measured variables between the groups (*P*>0.05). After treatment, there were significant differences in knee flexion ROM and WOMAC index (*P*<0.05) but no significant difference in knee extension ROM between the groups (*P*>0.05).

Conclusion: The addition of low-level laser therapy to a rehabilitation program post-TKR resulted in substantial enhancements in knee flexion range of motion and the WOMAC index.

## Introduction

The knee joint is one of the large joints in the body and is functionally of utmost importance for daily activities. This negatively affects the quality of life of patients with end-stage osteoarthritis (OA) [1]. Total knee replacement (TKR) is the best solution for patients with end-stage osteoarthritis, as it relieves joint pain and stiffness and improves mobility in 93% of cases [2]. Despite advances in implant design, surgical techniques, and knee kinematics, 20% of patients still experience limited joint activity, muscle atrophy, and pain after TKR, which negatively impacts activities of daily living [3,4]. Patients with TKR have persistent functional impairment, movement irregularities, and quadriceps weakness after surgery, potentially leading to long-term functional impairment after surgery [5]. Additionally, it is estimated that 3.48 million TKRs will be performed annually by 2030 [3].

The postoperative range of motion (ROM) of the knee is crucial to the patient's conviction after total knee arthroplasty (TKR). Patients with limited flexion angles or difficulty in activities before TKR show lower levels of belief compared to preoperative values [6]. Stiff knees are common in 8%-60% of patients with TKR [7]. Rehabilitation programs have been shown to be effective in restoring functional status and improving the clinical and social benefits of total knee replacement (TKR) [8]. However, there are no evidence-based guidelines for the best physical therapy protocol [9].

Low-level laser therapy is a non-invasive treatment for various musculoskeletal disorders that reduces swelling and pain and treats acute soft tissue injuries by modulating the inflammatory process [10]. LLL therapy significantly accelerates tissue healing by increasing collagen production, stimulating metabolic processes, regulating DNA synthesis, and improving nerve function [11,12]. It can also relieve chronic and acute knee pain, improve knee function, and improve overall balance [13]. The aim of this research was to determine the effects of LLL therapy on range of motion and functional abilities of the knee after TKR.

## Materials and methods

Design of the study and ethics statement

The study design utilized a randomized, double-blind, controlled trial. The Ethics Committee of the Faculty of Physiotherapy Research, Cairo University, approved the study protocol (Decision No. P.T.REC/012/002218). The research is listed in the Pan African Clinical Trials Registry under study ID PACTR201906789887048. No organization provided financial support for the research.

Sample size and participants

The sample size for the study groups was determined using the G* Power statistical tool (version 3.1) based on a previous study [6]. To ensure adequate statistical power for the present study, a predetermined power level of 0.95 and a significant level set at a p-value of 0.05 are used. This resulted in a sample size of 18 subjects in each group. However, a total of 58 participants were screened for eligibility, and 14 of them did not meet the inclusion criteria. Therefore, 22 participants were included in each group, taking possible dropouts into account.

Participants were recruited from the knee department of Mansoura University Hospital between June 2019 and December 2020. All patients signed informed consent after the benefits of the study and its procedures were explained.

Inclusion criteria

Patients with first-time TKR performed using the same operating technique (not more than two weeks after surgery) due to knee OA aged 50-70 years who were conscious, without cognitive impairment, could ambulate after surgery, with no other medical conditions other than osteoarthritis, and had a normal blood clotting index were included in the research.

Exclusion criteria

Patients with contraindications for LLL therapy, serious cardiovascular disease, neurological disease, hemophilia, severe diabetes, tumors, or coagulative disorders were excluded from the study.

Randomization

Before the trial began, a randomization table with a 1:1 allocation ratio was created using computer software. The allocation order was concealed by a series of sequentially numbered envelopes that were sealed opaquely to prevent the researcher and participants from knowing the allocation.

Interventions

Patients were seen at the end of the second week after operation and were randomly assigned to be subjected to either the intensive functional rehabilitation program alone (the control group) or LLL therapy in addition to the program (the experimental group). LLL therapy was applied twice per week for six weeks starting from day one of the third postoperative week around the knee at the incisional line, the medial and lateral intra-articular space, above and below the patella, and the popliteal fossa at low fluence (6 J/cm2, 650 nm, continuous wave) and 60 s per point with a total dose of 48 J per session. As previous studies indicated no agreement on the optimal duration of treatment, these doses and parameters of application were selected for use in knee OA [14].

The intensive functional rehabilitation program lasted 60-90 min each session and comprised a warm-up (5-10 min) of bilateral ankle dorsi-plantarflexion, alternate lower-limb flexion/extension, and stretching hamstrings; a specific strengthening program (15 min) of quadriceps isometric exercise in 0° and 60° flexion at visits 1-2, isometric 60° knee bending at visits 3-6, and hip abductors exercise in form of concentric/eccentric training at visits 1-4; a functional task-oriented program (15-20 min) of sitting down after getting up, quadriceps training from standing with a Thera band at visits 1-6, bilateral controlled squatting at visits 1-8, unilateral squatting to 90° at visits 7-10, ascending stairs and side-walking at visits 3-12, and upper-limb movements and walk in place with wide steps at visits 9-12; endurance exercises 15-20 min of walking at visits 3-12 and bicycling at visits 4-12; and cool-down (10 min) by walking slowly and cold packs on the knee joint [15]. All procedures were applied until the patient completed 12 treatment sessions in six weeks.

Outcomes

The primary outcomes were knee ROM (flexion and extension) and the level of functional abilities. Knee ROM was assessed with patients in a supine position using a digital goniometer (baseline Absolute Axis 0-185 Degrees Digital Goniometer©) and expressed in degrees, which is a reliable and valid method of evaluating joint ROM due to its high accuracy and eliminating the chance of errors by determining the "true" vertical or horizontal distance from a measurement's starting point [16].

The level of functional abilities was measured using the Arabic version of the Western Ontario and McMaster Universities Osteoarthritis (WOMAC) index, which has demonstrated good test-retest reliability (0.84, 0.84, and 0.92 for pain, stiffness, and physical function subscales, respectively) [17]. These assessments were made two weeks before commencing the physical therapy treatment postoperatively and again after six weeks of the physical therapy treatment program, with all measurement procedures performed by the same outcome assessor who was blinded to both groups.

Statistical analysis

Statistical Package for IBM Corp. Released 2013. IBM SPSS Statistics for Windows, Version 22.0. Armonk, NY: IBM Corp. was used for statistical analysis, and outcomes were considered statistically significant when p < 0.05. Descriptive statistical methods were used for the data analysis of all study variables (mean ± SD). Independent samples t-tests were used to test between groups, and paired sample t-tests were used to test within a group.

## Results

**Figure 1:**
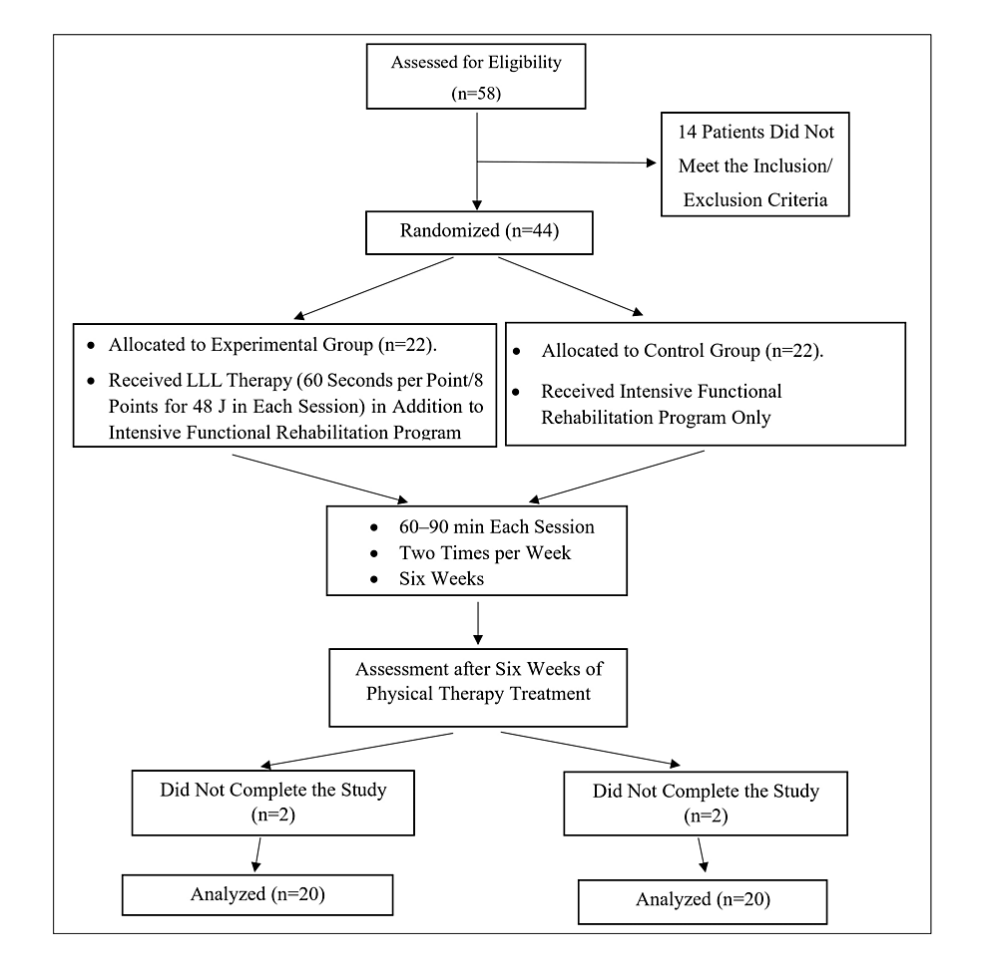
Flowchart for patient participation in the study LLL: Low-level laser

The study recruited 58 patients who underwent TKR surgery. From the total number, 44 were selected that met the inclusion criteria. They were randomly divided into control and experimental groups. Ultimately, 40 patients completed the study, 20 each, as two patients were excluded from the study due to travel. A flowchart for the participating patients is shown in Figure 1.

Table 1 lists the general characteristics of the patients: age, weight, and height. Table 2 presents the statistical clinical findings among the participating patients. Before the initiation of treatment, there was no statistically significant difference between the groups regarding general characteristics and clinical outcomes of all measurements for knee flexion and extension ROM and the WOMAC index for functional abilities (P > 0.05). In contrast, there was a statistically significant difference between groups post-application of the treatment program regarding knee flexion ROM and the WOMAC index (P < 0.05). No statistically significant differences were found between groups in knee-extension measurements (P > 0.05). There was a significant improvement in all variables within each group post-treatment.

**Table 1:** Comparing general characteristics between groups. P < 0.05 was considered statistically significant. SD: standard deviation; NS: not significant.

General Characteristic	Control Group n=20		Experimental Group n=20		P-value	Indication
	Mean	(±SD)	Mean	(±SD)		
Age (years)	60.85	(±4.158)	61.55	(±4.685)	(±0.620)	NS
Weight (kg)	90.55	(±6.815)	88.05	(±6.2025)	(±0.233)	NS
Height (cm)	166.78	(±6.367)	166.38	(±5.698)	(±0.835)	NS

**Table 2:** Outcome results of clinical characteristics pre- and post-treatment. Values are represented as mean±SD. P < 0.05 was considered statistically significant. ROM: range of motion.

Variable	Experimental Group n=20	Control Group n=20	P-value Between Groups
Flexion ROM			
Pre-treatment	46.70 (±6.562)	44.87 (±6.402)	0.379
Post-treatment	86.99 (±5.914)	68.41 (±2.821)	0.001
P-value within group	0.001	0.001	
Extension ROM			
Pre-treatment	11.29 (±1.550)	9.43 (±4.526)	0.089
Post-treatment	3.38 (±1.496)	3.60 (±2.039)	0.699
P-value within group	0.001	0.001	
WOMAC Index			
Pre-treatment	83.15 (±1.496)	82.75 (±1.372)	0.384
Post-treatment	28.15 (±6.426)	48.50 (±3.663)	0.001
P-value within group	0.001	0.001	

## Discussion

The current study was conducted to determine the effectiveness of adding LLLT therapy to a post-TKR rehabilitation program. Our results showed no statistically significant differences between groups in all pretreatment measurements (P > 0.05). However, after application of the treatment program, a statistically significant difference was observed between groups in knee flexion ROM and WOMAC index (P < 0.05), but there was no statistically significant difference between groups in knee extension measurements (P > 0.05).

After treatment, there was a significant improvement in all variables in both groups. LLL therapy, combined with an intensive functional rehabilitation program, demonstrated significant improvement in range of motion and functional abilities of the knee after TKR.

LLL therapy is a possible alternative to medical drugs in the treatment of arthritis because it can modulate the inflammatory process [18], increase fibroblastic activity and collagen production [19], increase lymphatic flow and reduce edema [20], and have a strong analgesic effect in relieving pain [12].

It is possible that LLL therapy improves knee range of motion after TKR by increasing collagen production by increasing the amount of hydroxyproline through the accumulation of ascorbic acid in fibroblasts, thereby increasing flexibility. In addition, LLL therapy leads to an increase in the proliferation of epithelial cells and fibroblasts [21].

After TKA, the range of motion for knee flexion still remains limited, which leads to functional impairments and patient dissatisfaction despite new surgical approaches, newer implants, and rehabilitation. Such stiffness is considered challenging for these patients and surgeons [22]. The results of the present study on knee range of motion are consistent with those of a previous study that concluded that patients required knee flexion of 67° for normal walking, 83° for stair climbing, and approximately 90° to 100° for stair climbing or standing and needed to get up from a sitting position on a chair. Therefore, TKR surgeons aim for knee flexion of 90° to 100° to minimize limitations in functional activities of daily living [23]. In the present study, these values were approximately achieved in almost all patients in the experimental group and in some patients in the control group.

A previous study found that postoperative flexion contracture results in weight shift and biomechanical abnormalities that negatively impact clinical outcomes [24]. In addition, up to 17% of TKR patients have a fixed flexion contracture after TKR [25]. After the physical therapy program, all patients in the current study had improved range of motion in knee flexion and extension.

The results of the present research are consistent with those of a previous study, which showed that starting rehabilitation within 24 hours of primary TKR resulted in improvement in knee flexion and extension after rehabilitation [25]. And this illustrates that ROM was improved after applying an intensive functional rehabilitation program in both groups, as in the current study. Likewise, our results were consistent with those of a study that examined the effectiveness of an intensive functional rehabilitation program in improving activities of daily living in patients with the first TKR. The results of the study showed that the program was effective in increasing the patients' performance in their everyday lives in the short and medium term [15].

Regarding the level of functional abilities, the WOMAC index is used as a self-assessment questionnaire to measure the level of pain, joint stiffness, and the ability to perform activities of daily living. LLL therapy has been shown to have a positive effect on postoperative pain [26], swelling [27], and range of motion [28].

Our results were supported by those of a previous study that reported that LLL therapy is a valid treatment method for restoring normality in various pathological conditions because it can relieve pain, accelerate wound healing, and improve tissue repair. Mechanistically, LLL therapy can reduce the severity of pain by lowering prostaglandin E2, inhibiting the onset of pain, and activating substances that control the inflammatory process [29]. Our results are also consistent with those of a study that examined the effectiveness of LLL therapy and exercise in the treatment of knee osteoarthritis using the WOMAC index to assess pain and functionality, showing good improvement on 24 questions in the WOMAC index stated [30]. Furthermore, our results agree with those of a meta-analysis and systematic review on the safety and effectiveness of LLL therapy in OA knee patients, which concluded that LLL therapy demonstrated significant differences in WOMAC score [31].

The current study, like any scientific study, has certain limitations. One of the major limitations is the presence of psychological factors that can influence the patient's reactions. The study's findings are based on a single geographic area and require further research to confirm them in other regions. Furthermore, there is no long-term follow-up to assess the long-term benefit of LLL therapy as an adjunct to physical therapy. Further studies are needed to evaluate other variables such as preoperative functional level, knee deformity, surgical approach, prosthesis type, and postoperative analgesics.

## Conclusions

LLL therapy offers important application possibilities in the area of postoperative physiotherapy. In the current study, both groups showed significant improvements in knee ROM and functional abilities after completing the intensive functional rehabilitation program alone or with the addition of LLL therapy to the program after TKR, with strong improvement in knee flexion ROM and the WOMAC index in the experimental group. Consequently, after TKR, knee flexion range of motion and WOMAC index are improved by incorporating LLL therapy into the rehabilitation program.
